# Are American Indian/Alaska Native Adolescent Health Behaviors Different? A Review of AI/AN Youth Involved in Native STAND Curriculum, 2014–2017 United States

**DOI:** 10.1007/s10995-021-03256-7

**Published:** 2021-10-27

**Authors:** Megan Skye, Stephanie Craig, Caitlin Donald, Allyson Kelley, Brittany Morgan, Kavita Rajani, Michelle Singer, Tosha Zaback, William Lambert

**Affiliations:** 1 Oregon Health and Science University, 3181 SW Sam Jackson Park Rd., Portland, OR 97239 USA; 2 Northwest Portland Area Indian Health Board, Portland, OR 97201 USA; 3 Allyson Kelley and Associates, Sisters, OR 97759 USA

**Keywords:** American Indian Alaska Native youth, Health profiles, Culture-based curriculum

## Abstract

**Objectives:**

To explore health behavior profiles of AI/AN youth involved in native students together against negative decisions (STAND), a national culture-based curriculum.

**Methods:**

We analyzed data from 1236 surveys conducted among AI/AN youth at 40 native STAND implementation sites located in 16 states throughout the US from 2014 to 2017. Health profiles included demographics, sexual orientation, sexual activity, STI testing, cigarette use, and suicide attempts in the past 12-months. We used t-tests and chi square tests of independence to compare risk behavior prevalence among the sample.

**Results:**

Health behavior profiles of AI/AN youth indicate that 45.6% of youth did not use condoms the last time they had sex, and 82.7% have never been tested for STIs. Differences in cigarette smoking were observed in questioning youth (questioning: 80.3%, straight/heterosexual: 63.8%, LGBTQ2S + : 49.9%, p = 0.03).

**Conclusions for Practice:**

Health behaviors related to sex, substance, violence and self-harm, are at least as common for AI/AN youth as those observed in other US teens. Future research should consider similarities and differences in health profiles of AI/AN youth when designing interventions that affect them. Further, our findings underscore the need for culturally-relevant curricula like native STAND, not because their health behavior is different, but because their socio-ecologic environment is different.

**Supplementary Information:**

The online version contains supplementary material available at 10.1007/s10995-021-03256-7.

## Significance Statement

This paper demonstrated that AI/AN adolescent health behaviors related to sex, substance misuse, violence, and self-harm are at least as common among AI/AN youth as those observed in other US teens. The biggest thing that this paper adds to the current knowledge is a new idea that AI/AN youth health behaviors may not be that different than other youth in the US. The fact that AI/AN youth health trajectories are much different from other populations (for example shorter life expectancy) calls attention to future research that explores what happens to AI/AN youth as they age into adulthood.

## Introduction

During adolescence, risky health behaviors, such as early sexual debut or alcohol and drug misuse, are associated with negative outcomes that track into adulthood (McCabe et al., 2016; Spriggs & Halpern, 2008a); Springs & Halpern, 2008b. While much of this research has described youth’s health behaviors in the general population, very little research has focused on the behaviors of American Indian and Alaska Native (AI/AN) youth. The available data is frequently limited in scope; results are often from a small number of communities or residential Bureau of Indian Education (BIE) schools (Beauvais et al., 2008; Blum et al., 1992; Borowsky et al., 1999; Whitesell et al., 2007). The results from these studies have nevertheless provided valuable insights into the prevalence of AI/AN youth’s behavioral health. Subica and Wu report that AI/AN youth experience higher rates of illicit substance use, depressed moods, and suicidality (Subica & Wu 2018). A 2015 report published by the Indian Health Service (IHS) found that AI/AN adolescent and young adult suicide rates are up to six times greater than non-AI/AN populations (US Department of Health and Human Services [USDHHS], 2017). Beauvais et al. found that over half of AI/AN youth reported using tobacco in 2005 (2008). Studies with larger sample sizes have echoed these results. Stanley et al. compared substance use patterns in 1399 AI/AN youth to national patterns from a Monitoring the Future Study, and results revealed that Native youth had higher levels of substance use, particularly marijuana (Stanley et al., 2014). National data from the Youth Risk Behavioral Study (YRBS) from 2007–2009 found that AI/AN youth had higher odds of engaging in sexual risk-related health behaviors than their white peers (USDHHS, 2020).

The health behaviors for Native youth are not monolithic; however, there are regional variations in behavior across Native communities (de Ravello et al., 2014; Wingo et al., 2012). In one study, Northern Plains and Upper Great Lakes American Indian (AI) youth were more likely to have used substances and at much higher rates than AI youth living in the Southern Plains (Miller et al., 2012). AI youth living in urban settings might be more vulnerable to substance use than AI youth living in rural and reservation settings (Kulis et al., 2016). LaFromboise and Dizon examined risk-taking in urban AI youth and found that the lack of cultural ties and kinship systems experienced by some urban AI youth might contribute to risky health behaviors (2003). Access to cultural activities and connections has also been shown to be protective. Markham and colleagues found that cultural connection can help AI/AN youth delay sexual activity (2015). Borowsky et al. found that while AI/AN youth had the highest rates of suicide of any ethnic group in the United States, community and family connections were protective against suicide attempts (1999).

Documenting youth’s unique health behaviors is necessary for developing tailored interventions that address risk factors while building on strengths. Further, interventions and curricula designed for AI/AN youth, guided by culture and values, are now being used by some tribal communities to promote healthy decision-making and build lifelong resilience (Kelley et al., 2015, 2018; Lafromboise & Lewis, 2008).

Culturally adapted interventions are emerging as an effective way to address AI/AN youth’s unique health behaviors and worldviews. One example of an adapted culture-based curriculum is native students together against negative decisions (STAND) (Smith & Rushing, 2011). Native STAND is a 29-session curriculum based on the STAND intervention, which was designed and evaluated among rural youth in the southern United States and found to promote condom self-efficacy, STI risk behavior knowledge, and conversations with peers about other sexual health topics among participating students. In 2008, Native STAND was adapted by a national group of AI/AN partners and topical experts to be culturally relevant for AI/AN youth. Between 2009–2010, Native STAND was piloted at four Bureau of Indian Education (BIE) boarding schools throughout the United States, reaching 70 AI/AN youth in the 10^th^ grade. Evaluation results from BIE schools demonstrated significant improvements in knowledge of STI/HIV prevention, reproductive health, and healthy relationships (Smith & Rushing, 2011). Native STAND was then implemented with 90 AI/AN students at a tribal high school from 2013 to 2014. This study found positive results, with increases in confidence, self-esteem, and youth involvement in culture and community (Rushing et al., 2017). Previous Native STAND reports show promise, but more research is needed to classify it as an evidence-based curriculum. This present study uses baseline wave data from Native STAND to report behavioral health profiles for 1236 AI/AN youth involved in the program, living across the U.S.

The multisite study was carried out in partnership with the Prevention Research Center at Oregon Health & Science University (OHSU), the Northwest Portland Area Indian Health Board (NPAIHB), and 48 Tribes and Tribal organizations who enrolled in the study. The NPAIHB is a Tribal organization that represents 43 federally recognized Tribes in Washington, Oregon, and Idaho. The mission of the NPAIHB is to “eliminate health disparities and improve the quality of life of American Indians and Alaska Natives by supporting Northwest Tribes in their delivery of culturally appropriate, high-quality health care.” The NPAIHB’s governing board meets quarterly and is composed of one delegate from each member Tribe, selected by the individual Tribal governments. The Northwest Tribal Epidemiology Center is housed under NPAIHB and provides support in the way of research, surveillance, and public health capacity building in partnership with the Northwest Tribes. Primary IRB approval for this study was granted by the Portland Area Indian Health Service Institutional Review Board in Portland, Oregon. IRB documents with their approval were sent to the IRB at Oregon Health Sciences University for their records. All instruments were reviewed and approved by the IRB before data collection took place.

Consistent with a culturally-centered, health equity approach, our Native STAND team and authors of this manuscript include members of 2 American Indian and Alaska Native tribes, 1 sexual minority group, 8 gender minorities in research, all have been historically underrepresented in research and publications.

## Methods

### Study Design

**Fig. 1:**
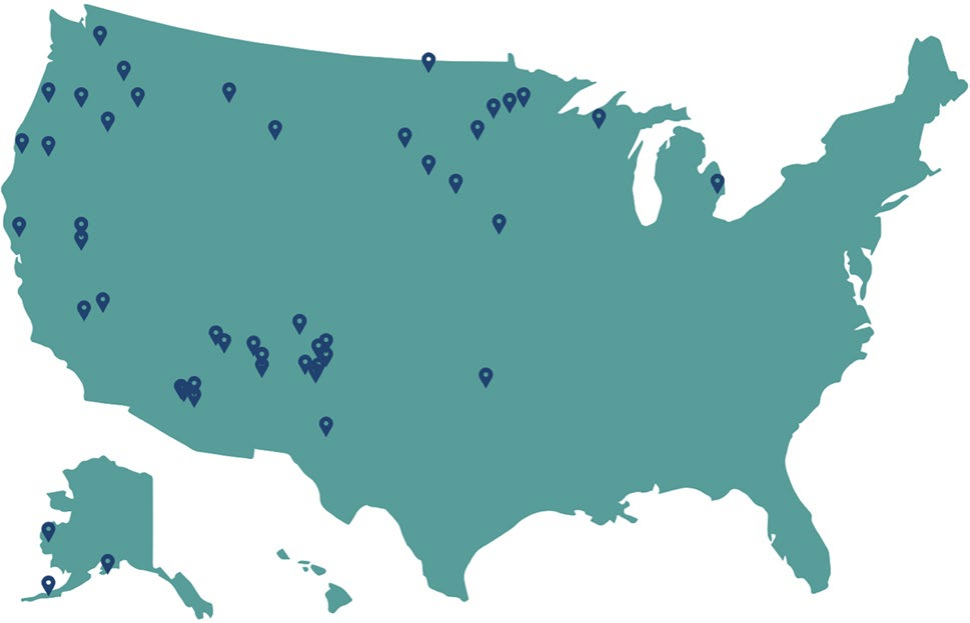
Geographical reach of the native STAND project

From November 2014 to March 2017, we recruited Tribes and youth-serving organizations in Indian Country and Alaska to join one of three cohorts of sites who participated in the study. At the end of the recruitment period, a convenience sample of 48 sites, selected an educator to attend an all-expense paid week-long training on the Native STAND program in Portland, OR. After training, we monitored the participating sites and educators for two years using monthly fidelity calls and annual interviews. Sites received a modest stipend of $5000 per year to offset the cost of implementation (Fig. 1).

### Study Sites and Participants

The participating educators delivered the Native STAND curriculum in a variety of in-school and after-school settings. The study sites were located in 16 states, including Alaska. Most of the sites were from rural communities (36/48, 75%), and were from the western part of the United States (36/48, 75%). As of March 2019, 40/48 (85%) of the trained sites had completed at least one round of implementation, with a total of 90 completed implementations across the sites.

Most of the 40 sites that delivered the curriculum reported that they restricted their recruitment to a specific school’s student body (32/40 sites, 80%), with the rest reporting they recruited from their larger communities. A majority of the sites (30/40 sites, 75%) also reported that their classes occurred outside of school hours, and therefore their enrollment in the Native STAND was voluntary. Some sites were given permission to incorporate Native STAND into their physical or health education curriculum during the school day; and offer students class credit for attending the lessons (10 sites, 25%), which lowered the barriers for youth to participate in Native STAND.

The youth who assented to participate in Native STAND filled out a baseline survey prior to participation, from September 2015 to March 2019. We included all of the youth who filled out a baseline survey in this analysis, except for youth who did not identify as AI/AN (n = 220).

### Data Collection and Management

We collected baseline measures via paper survey from youth who returned signed parental consent and personal assent forms to their site educator before starting Native STAND lessons. The survey assessed a broad range of health knowledge, attitudes, beliefs, intentions, behaviors, and skills relating to physical, sexual, mental, and psycho-social health. Each youth received a paper survey labeled with a unique study ID and a Business Reply Mail manila envelope to ensure confidentiality of survey responses. Upon completing the survey, youth sealed the envelope and returned it to their educator, who then mailed it to the Native STAND project office. Once received at the project office, individual youth data was not shared with site educators.

Once collected, we managed our data with REDCap electronic data capture tools hosted at Oregon Health & Science University (Harris et al., 2009). REDCap (Research Electronic Data Capture) is a secure, web-based application designed to provide complete data capture and management support for research studies. Survey data was analyzed using Stata, version 15.0 (StataCorp, College Station, TX, USA). We discussed findings with Native STAND site educators and community leaders before submission to ensure the findings corroborated with the communities’ experiences.

### Measures

Our baseline survey collected demographic information including age, race/ethnicity, gender (girl, boy, transgender), sexual orientation (straight/heterosexual, lesbian, gay, bisexual, transgender, queer, two-spirit + (LGBTQ2S +), unsure/questioning), and community location (urban communities were those in urbanized area as defined by the U.S. census, and rural communities were those outside of an urbanized area). We also collected information about sexual activity, including lifetime history of oral sex and vaginal sex, and sexually transmitted infection (STI) testing. We also asked youth who reported a history of vaginal sex the age of their vaginal sexual initiation and whether they used condoms during their last vaginal sexual encounter. In addition to sexual health information, we asked youth about their lifetime history with smoking tobacco cigarettes, and if youth had attempted suicide in the past year.

### Statistical Analyses

Before conducting analyses, we carried out a sensitivity test to identify differences in behavior by the number of youth participating in the site (a large site was defined as a site with more than 100 participants (2/48 sites (4.2%), 415 youth (33.6%)), a small site was defined as a site with 100 or fewer participants (46/48 sites (95.8%), 821 youth, 66.4%)). Statistically significant differences were detected between the site-size subgroups in the prevalence of vaginal sex (large: 19.5%, small: 31.8%, p < 0.001), oral sex (large: 13.6%, small: 23.5%, p < 0.001), and condom use (large: 12.2%, small: 15.7%, p < 0.001). Analyses therefore weighted participant responses by their site size using inverse probability weights with Stata’s SVY (survey data) commands. We conducted another sensitivity analysis comparing the behavior of youth from rural and urban communities. No significant differences were found, with the exception of STI testing rates (rural: 7.6%, urban: 21.9%, p = 0.007). Therefore, results were not weighted or stratified based on community location. We used weighted chi-square tests of independence to compare health behavior among subgroups for all categorical variables and weighted T-tests to test for differences in age of vaginal sexual initiation among subgroups. The goal of this analysis was to present a description of the study population’s behavior profile, so we did not conduct any multivariate analyses.

The Youth Behavioral Risk Survey (USDHHS, 2017) has reported differences in health behavior by age and gender, so we examined differences between boys and girls of 14 and younger, and differences between boys and girls 15 and older. We chose to split the sample at this age group, as 15 is a common age in which youth start high school, and because the average age of vaginal sexual initiation of the sample was approximately 14 (see Table 1). We only included youth who identified as girls or boys in this cross-section of the sample population, as our sample contained only 16 youth who identified as transgender.

**Table 1:** Demographics and health behaviors of American Indian/Alaska Native youth n = 1236

	Unadjusted	Adjusted*(%)
	% (n)	%
Age		
14 and under	45.2% (556)	40.6%
15 and over	54.8% (673)	59.4%
Gender		
Girl	52.3% (643)	58.7%
Boy	46.5% (572)	40.1%
Transgender	1.2% (15)	1.2%
Sexual orientation		
Straight/heterosexual	77.3% (924)	79.6%
LGBTQ2S +	9.3% (111)	8.6%
Unsure/questioning	13.4% (161)	11.8%
Community location		
Urban	17.9% (221)	24.7%
Rural	82.1% (1,015)	75.3%
Ever had vaginal sex		
Yes	27.6% (310)	30.3%
No	64.6% (726)	61.1%
Decline to answer	7.8% (88)	8.6%
Ever had oral sex		
Yes	20.1% (228)	19.4%
No	70.4% (799)	70.7%
Decline to answer	9.5% (108)	9.9%
Age of sexual initiation (M, SD)	14.14 (1.74)	14.00 (1.74)
Used condom last time having vag. sex**		
Yes	48.7% (150)	42.7%
No	41.6% (128)	45.6%
Decline to answer	9.7% (30)	11.7%
Ever been tested for STIs		
Yes	10.7% (122)	11.1%
No	80.9% (925)	82.7%
Don’t know	4.4% (51)	3.6%
Decline to answer	4.0% (46)	2.6%
Ever used cigarettes		
Yes	35.6% (139)	35.4%
No	64.4% (956)	64.6%
Attempted suicide in last 12 months		
Yes	12.5% (139)	10.6%
No	86.3% (956)	88.6%
Decline to answer	1.2% (13)	0.7%

Unadjusted variable denominators differ depending on missingness. Variables missing at 10% or less

*Proportions from population estimates based on inverse frequency weighting by site size

**For youth who answered “Yes” to having had vaginal sex (n = 308)

Differences in health behavior and health outcomes have been found between youth with different sexual orientations in several studies, including the 2017 Youth Behavioral Risk Survey [USDHHS, 2017; Marshal et al., 2012; Rosario et al., 2014)]. We, therefore, examined between-group differences between youth who identified as straight/heterosexual, LGBTQ2S + , and unsure/questioning.

## Results

### Participants

Our sample included 1236 AI/AN youth. Our sample contained more boys (52.3%) than girls (46.5%), with 1.2% of youth identifying as transgender (see Table 1). Most of our sample was age 15 or over (59%), with a mean age of 14.88 (SD: 1.68). Most of the youth identified as straight/heterosexual (77.3%), although 9.3% identified as LGBTQ2S + , and 13.4% reported unsure/questioning their orientation. Sites ranged in size from 2 to 310 youth (M = 107.5, SD = 115.48).

After adjusting for site sample size, we found that a majority of youth (82.7%) reported never being tested for STIs. Over a third of youth reported lifetime tobacco cigarette use (35.4%), and 10.6% of youth reported attempting suicide in the last 12 months. We found that a majority of our youth had not engaged in vaginal sex (61.1%) or oral sex (70.7%). For those youth who had engaged in vaginal sex, 45.6% of those youth did not use a condom the last time they had sex.

### Differences by Gender, Stratified by Age

The reported health behavior for younger boys and girls were different from those reported by older boys and girls. For the younger age group, girls and boys reported similar health behaviors, with the exception of suicide attempts. Younger girls were more likely to report a recent attempt than younger boys, although these differences were only marginally significant (younger girls: 17.2%, younger boys: 10.5%, p = 0.09) (see Table 2). In the older age group, we observed more differences. Not only were older girls more likely to report a recent suicide attempt than older boys (girls: 10.0%, boys: 5.1%, p = 0.19), they also more commonly reported STI testing (girls: 18.1%, boys: 8.3%, p = 0.02). We also observed differences in condom use for older boys and girls, with older girls less likely to report using condoms than older boys (older girls: 42.3%, older boys: 53.4%, p = 0.03) (Supplemental Fig. 1).

**Table 2:** Health behaviors of AI/AN youth by age group and gender

	Gender—proportions*(%)		
	Girls	Boys	P value**
*Age group: 14 and younger*			
Vaginal sex			0.4
Yes	17.8%	14.3%	
No	79.0%	77.9%	
Decline to answer	3.2%	7.8%	
Oral sex			0.98
Yes	9.7%	9.9%	
No	85.1%	84.3%	
Decline to answer	5.2%	5.8%	
Age of sexual initiation (M, SD)	13.28 (0.69)	12.97 (1.29)	0.36
Used condom last time having vag. sex***			0.64
Yes	29.7%	27.0%	
No	68.3%	65.5%	
Decline to answer	2.0%	7.5%	
Ever been tested for STIs			0.77
Yes	6.9%	6.1%	
No	87.6%	86.2%	
Don’t know	2.2%	4.3%	
Decline to answer	3.4%	3.4%	
Ever used cigarettes			0.58
Yes	23.1%	26.6%	
No	76.9%	73.4%	
Attempted suicide in last 12 months			0.09
Yes	17.2%	10.5%	
No	82.8%	88.9%	
Decline to answer	0%	0.6%	
*Age group: 15 and older*			
Vaginal sex			0.74
Yes	41.7%	37.5%	
No	48.7%	51.7%	
Decline to answer	9.6%	10.7%	
Oral sex			0.58
Yes	24.6%	28%	
No	64.3%	58.7%	
Decline to answer	11.1%	13.3%	
Age of sexual initiation (M, SD)	14.36 (1.52)	13.98 (2.42)	0.41
Used condom last time having vag. sex***			0.03
Yes	42.3%	53.4%	
No	48.8%	24.0%	
Decline to answer	8.9%	22.6%	
Ever been tested for STIs			0.02
Yes	18.1%	8.3%	
No	77.7%	83.8%	
Don’t know	2.7%	5.6%	
Decline to answer	1.5%	2.3%	
Ever used cigarettes			0.54
Yes	41.3%	45.7%	
No	58.7%	54.3%	
Attempted suicide in last 12 months			0.19
Yes	10.0%	5.1%	
No	89.0%	94.1%	
Decline to answer	1.0%	0.8%	

*Proportions from population estimates based on inverse frequency weighting by site size

**P values from t-tests or chi-squares utilizing inverse frequency weighting

***For youth who answered “Yes” to having had vaginal sex

Differences in reported health behavior were also observed in relation to sexual orientation (see Table 3). Heterosexual and LGBTQ2S + youth had similar prevalence of most health behaviors; we observed most differences in questioning youth. Fewer questioning youth reported engaging in both vaginal (questioning: 12.8%, straight/heterosexual: 32.6%, LGBTQ2S + : 35.6%, p = 0.03) and oral sex (questioning: 7.5%, straight/heterosexual: 20.5%, LGBTQ2S + : 27.7%, p = 0.11). They also more commonly reported using condoms during vaginal sexual activity (questioning: 56.0%, straight/heterosexual: 40.7%, LGBTQ2S + : 50.5%, p = 0.11) (Supplemental Fig. 1). However, questioning youth on average initiated vaginal sex earlier than their heterosexual and LGBTQ2S + counterparts (questioning: 12.30 years, Straight/Heterosexual: 14.05 years, LGBTQ2S + : 13.83 years, p = 0.07). In terms of cigarette smoking, differences were again observed in questioning youth, with fewer of them reporting ever smoking cigarettes (questioning: 19.7%, straight/heterosexual: 36.2%, LGBTQ2S + : 50.1%, p = 0.03).

**Table 3:** Health behaviors of AI/AN youth by sexual orientation

	Proportions*(%)			
	Straight/heterosexual	LGBTQ2S +	Unsure/questioning	P value**
Vaginal sex				0.03
Yes	32.6%	35.6%	12.8%	
No	58.1%	61.3%	78.6%	
Decline to answer	9.3%	3.1%	8.6%	
Oral sex				
Yes	20.5%	27.7%	7.5%	0.11
No	69.3%	65%	83%	
Decline to answer	10.2%	7.3%	9.5%	
Age of vaginal sexual initiation (M, SD)	14.05 (1.66)	13.83 (1.03)	12.30 (2.94)	0.07
Used condom last time having vag. sex***				0.11
Yes	40.7%	50.5%	56.0%	
No	47.9%	44.6%	15.5%	
Decline to answer	11.4%	4.9%	28.5%	
Ever been tested for STIs				0.04
Yes	83.6%	76.6%	81.8%	
No	10.5%	21.9%	7.6%	
Don’t know	3.7%	1.5%	3.9%	
Decline to answer	2.1%	0%	6.6%	
Ever used cigarettes				0.03
Yes	36.2%	50.1%	19.7%	
No	63.8%	49.9%	80.3%	
Attempted suicide in last 12 months				< .001*
Yes	7.5%	27.3%	20.6%	
No	91.8%	71.8%	78.6%	
Decline to answer	0.7%	0.9%	0.9%	

*Proportions from population estimates based on inverse frequency weighting by site size

**P values from t-tests or chi-squares utilizing inverse frequency weighting

***For youth who answered “Yes” to having had vaginal sex

Differences were also observed with LGBTQ2S + youth. More LGBTQ2S + youth reported a recent suicide attempt, compared to both their heterosexual and questioning peers (LGBTQ2S + : 27.3%, straight/heterosexual: 7.5%, questioning: 20.6%, p < 0.001).

## Discussion

The Native STAND baseline data confirms that health behaviors related to sex, substance misuse, violence, and self-harm, are at least as common for AI/AN youth as for other US teens.

Our sample closely followed trends observed in the 2017 YRBS in relation to condom use and STI testing, with more boys than girls reporting using condoms and more girls than boys reporting being tested for STIs. The prevalence of recent suicide attempts in our youth participants was similar to prevalence reported in the 2017 YRBS and in previous Native STAND interventions (Rushing et al., 2017) with more girls than boys reporting a recent attempt, and more LGBTQ2S + reporting an attempt than Heterosexual and Questioning youth (USDHHS, 2017). Some studies have indicated that Native youth are more vulnerable than other youth, (de Ravello et al., 2014; Everett et al., 2000; Kaufman et al., 2007). Our data indicates that while AI/AN youth are susceptible to risky health behaviors, their profile might not be that different from their White, Latinx, and African-American peers.

One exception is the reported prevalence of cigarette smoking among Native STAND participants. The prevalence was higher than the prevalence reported for any racial/ethnic group surveyed in the 2017 YRBS (White youth: 31%, Black youth: 21%, Latinx youth: 21%) (Kann et al., 2018). Other studies (Beauvais et al., 2008) and the BIE Native STAND intervention cohort showed similar results, with 88.2% of participants reporting lifetime cigarette use (Smith & Rushing, 2011). The confirmation of this finding in our study indicates that this potential issue requires further scrutiny into how much of the high smoking prevalence is due to ceremonial as opposed to commercial use of tobacco, and why youth questioning their sexual orientation had a lower prevalence of cigarette smoking. Cigarette smoking is not the only difference that was observed for questioning youth. Questioning youth in our program were less likely to be sexually active and reported higher levels of condom usage when engaging in vaginal sex. Despite these indications that youth questioning their sexual orientation might be less vulnerable to risky health behaviors, these youth nonetheless reported a lower average age of sexual initiation than their LGBTQ2S + and Heterosexual peers.

Research on sexual orientation and gender identity formation is limited (Bosse, 2019). Most models and theories are based on non-AI/AN populations and adults in the US populations, and these fail to take into account the cultural aspects of identity and historically how AI/AN Tribes viewed the identities and unique roles of LGBTQ2s + Tribal members. In general, research about youth has indicated that earlier sexual debuts might be associated with depressive symptomology (Felner et al., 2021; Spriggs & Halpern, 2008a, 2008b, 2008c). It is possible that if a youth has had an early sexual debut, and the experience is negative, it might cause a divergence from the common trajectory of youth exploration and development, leading to the lower levels of the health behaviors we observed in these youth.

Native STAND findings are among the first to provide insight into risk profiles of AI/AN youth based LGBTQ2S + and gender. National studies like the YRBS do not differentiate risk profiles based on LGBTQ2S + or racial and ethnic classification. Future national surveys may consider what makes youth at risk for poor health outcomes, and what youth need to thrive.

### Limitations

Although many students from diverse communities participated in the Native STAND curriculum, we are unable to generalize these results to Native youth as a whole because we did not have a probabilistic sample of youth. Youth who agreed to participate in the Native STAND curriculum may have had different behavior profiles than other youth, as each Native STAND community utilized different recruitment strategies for their youth cohorts. Certain communities could have selected youth to participate because they were seen as being more at-risk, and therefore these youth might have more elevated levels of risky health behavior than other youth in their community. For other communities where enrollment in Native STAND was voluntary, the youth that chose to participate might already have been interested in healthy decision-making, with a lower level of risk than other youth in their community. The final results we see in this analysis may be confounded by these competing recruitment strategies.

We are also limited in our ability to generalize our findings to the United States as a whole due to the limited representation from the Northeast and Southeastern Tribes. Another limitation is that our results rely on data from baseline surveys. As many of the survey questions were sensitive in nature, youth may have been more likely to skip or alter their responses in this first survey due to a lack of trust in the confidentiality of the Native STAND project (Furlong et al., 2004). This response bias might have been exacerbated in sites with fewer students, where students could have felt less secure in their anonymity, and therefore shared less sensitive information. Of particular concern is how this bias might have affected the reporting of sexual orientation. There may be underreporting of the number of youth identifying as LGBTQ2S + and questioning.

A further limitation is the mischievous responses from participants that potentially distort results. Fan and colleagues found that these responses were most likely when adolescents were given self-administered questionnaires that had unusual or exceptional response options (Fan et al., 2006). Youth may have over-reported their prevalence of risky health behaviors and have over-reported identifying as LGBTQ2S + and/or Questioning (Fish & Russell, 2018). Future research involving the Native STAND curriculum will test for mischievous responses.

Finally, while our baseline survey captured a lot of information on common adolescent health behavior, this survey did not capture extensive demographic information about the youth’s communities. Culturally-relevant curricula like Native STAND may be necessary for Native youth, not because their health behavior is different, but because their socio-ecologic environment is different. AI/AN communities often rely more heavily on cultural activities, language, traditions than non-Native communities, and need curricula that reflect and celebrate these differences (Thornton et al., 2016).

### Public Health Implications

Future publications will examine differences in youth behavior in their pre- and post- surveys from Native STAND.

Examining the context surrounding cigarette smoking for tribal youth is an important next-step for adolescent health advocates. Questionnaires looking at cigarette use in Native youth should clearly distinguish between commercial and ceremonial use and now vaping. Future studies should also focus on the vulnerabilities and strengths of Native youth as they develop their sexual and gender identities and explore how recruitment strategies affect the baseline behavior of participating youth. Documenting how culturally-adapted interventions address multiple health behaviors and explore effectiveness based on risk conditions is an essential next step in assessing curricula like Native STAND (Wright et al., 2020). Exploring connections between knowledge, behavior, self-efficacy, and community conditions are necessary. Health behaviors are often cumulative and dynamic, based on a complex interplay of family, community, culture, and socio-economic factors.

To examine differences observed for questioning youth more completely, a study tracking AI/AN youth through adolescence might shed light on the relationship between sexual identity development and health behavior. In sum, documenting AI/AN youth’s baseline health profiles is essential when developing or adapting interventions designed with this population in mind.

## Supplementary Information

Below is the link to the electronic supplementary material.

Supplementary file1 (DOCX 55 kb)
