# Supplementary figures and images for: Are American Indian/Alaska Native Adolescent Health Behaviors Different? A Review of AI/AN Youth Involved in Native STAND Curriculum, 2014–2017 United States
Source: Matern Child Health J. 2021 Oct 27;25(12):1893–902. doi: 10.1007/s10995-021-03256-7 (PMC8599210; doi:10.1007/s10995-021-03256-7)

Supplemental Figure 1: Condom Usage by Gender and Age Group, Sexual Orientation


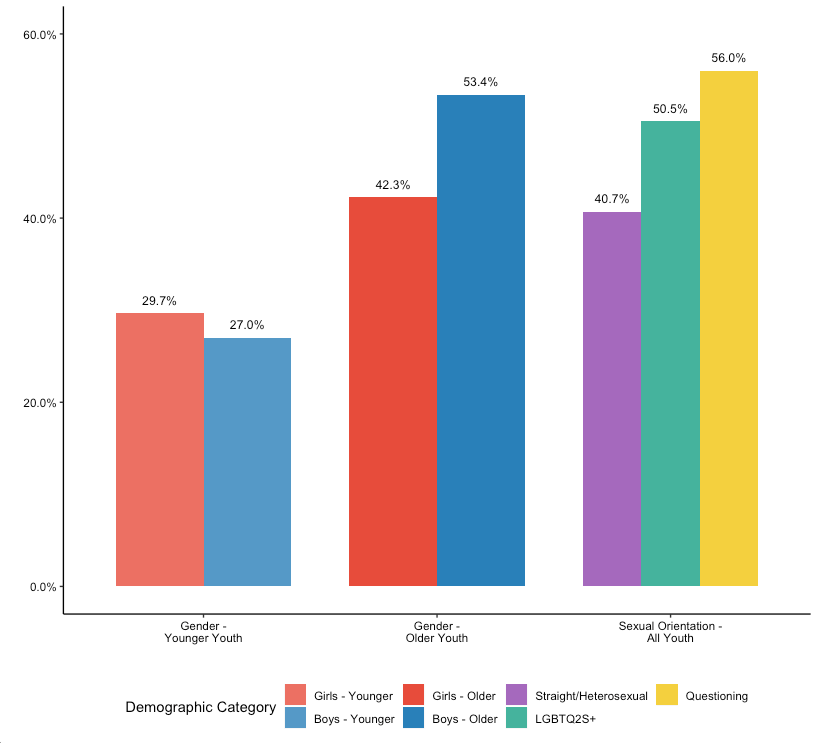

Supplement: Supplementary file 1 — Supplementary file1 (DOCX 55 kb) [file 10995_2021_3256_MOESM1_ESM.docx]
